# Lower extremity joint contracture according to ambulatory status in children with Duchenne muscular dystrophy

**DOI:** 10.1186/s12891-018-2212-6

**Published:** 2018-08-16

**Authors:** Young-Ah Choi, Seong-Min Chun, Yale Kim, Hyung-Ik Shin

**Affiliations:** 1 Department of Rehabilitation Medicine, National Traffic Injury Rehabilitation Hospital, 260, Jungang-ro, Yangpyeong-eup, Yangpyeong-gun, Gyeonggi-do 12564 Republic of Korea; 2 Department of Rehabilitation Medicine, Purme Foundation NEXON Children’s Rehabilitation Hospital, 494 World-Cup Buk-ro, Mapo-gu, Seoul, 03918 Republic of Korea; 3 Department of Rehabilitation Medicine, Seoul National University Hospital, 101 Daehak-ro, Jongno-gu, Seoul, 03080 Republic of Korea

**Keywords:** Neuromuscular disease, Duchenne muscular dystrophy, Corticosteroid, Contracture, Stretching

## Abstract

**Background:**

Lower extremity joint contractures have negative effects on gait in children with Duchenne muscular dystrophy (DMD). Thus, contracture prevention is essential for maintaining a patient’s functional ability and an acceptable quality of life. This study investigated hip flexion (HF), knee flexion (KF), and ankle joint plantar flexion (APF) contractures among male patients with DMD, based on the patients’ ambulatory status. Differences in major joint contractures, based on passive stretching exercise participation, were also investigated.

**Methods:**

A total of 128 boys with DMD, followed at the DMD clinic of a tertiary care hospital, were included in this cross-sectional study. The passive ranges-of-motion of the hip, knee, and ankle joints were measured, in the sagittal plane, using a goniometer. The Vignos Scale was used to grade ambulatory function. Boys with DMD who performed stretching exercises for more than 5 min/session, > 3 sessions/week, were classified into the stretching group.

**Results:**

The HF (23.5^o^), KF (43.5^o^), and APF (34.5^o^) contracture angles in the non-ambulatory group were more severe than those in the ambulatory group. APF contractures (41 patients, 52.6%) were more frequently observed early, even within the ambulatory period, than were hip (8 patients, 10.3%), and knee joint (17 patients, 21.8%) contractures. Passive stretching exercises > 3 sessions/week were not associated with the degree of lower extremity joint contractures in the ambulatory or non-ambulatory group.

**Conclusion:**

HF, KF, and APF contractures are more common and severe when there is deterioration of ambulatory function. Stretching exercises alone are unlikely to prevent lower extremity joint contractures.

## Background

Duchenne muscular dystrophy (DMD) is an X-linked recessive disorder caused by a lack of dystrophin, and has an overall incidence of 1 in 4700 male births [1, 2]. Muscular dystrophy is characterized by progressive muscular weakness, from childhood, which eventually results in the loss of gait performance when patients are approximately 10–12-years-old [3].

In patients with neuromuscular conditions, contractures develop due to intrinsic myotendinous structural changes and extrinsic factors [4]. Specifically, in patients with DMD, joint contractures are associated with several factors, including loss of full joint range-of-motion (ROM), static positioning, muscle imbalance around a joint, and fibrotic changes (fatty tissue infiltration) within muscle tissues [5]. Lower extremity joint contractures negatively affect the gait of patients with ambulatory DMD [6]. For example, hip joint contractures can lead to pelvic obliquity, which is associated with scoliosis development [7]. In recent decades, the survival of patients with DMD has improved because of interdisciplinary care, such as the inclusion of noninvasive ventilation [8]. Hence, contracture prevention is essential for maintaining a patient’s functional ability and an acceptable quality of life.

Although knowledge regarding lower extremity joint contracture profiles, based on disease progression, is necessary for the development of appropriate preventive strategies, studies is this area remain scarce. McDonald et al. [9] reported that lower extremity contractures were rare in patients able to maintain an upright posture, but developed soon after the patients became confined to a wheelchair for the majority of the day. However, these authors did not describe any differences between the major lower extremity joints, i.e., the hip, knee, and ankle. This study aimed to investigate the profile of hip, knee, and ankle joint contractures, based on the ambulatory status of patients with DMD. Furthermore, differences in major joint contractures were evaluated, based on the passive stretching exercises performed by ambulatory and non-ambulatory patients.

## Methods

### Participants

Overall, 136 boys with DMD were included in this cross-sectional study conducted at the DMD clinic of Seoul National University Hospital (Seoul, Korea). DMD diagnoses were confirmed using a dystrophin gene study. The genetic test methods used to identify dystrophin mutations were multiplex polymerase chain reaction and direct sequencing (Xp21.2-p21.1, exons 1–79). If the deletion/duplication testing results were negative, dystrophin gene sequencing was performed to search for point mutations or small deletions/insertions. All children with DMD participating in the study were prescribed alternate-day deflazacort (0.9 mg/kg), according to the international consensus, after demonstrating a partial Gower sign [10]. Patients were excluded from the study due to the presence of co-morbidities (e.g., an acquired brain or spinal injury), absence of corticosteroid administration, use of ankle-foot orthosis (AFO), or use of therapeutic weight bearing (e.g., passive standing frame) to increase lower extremity ROM. The research was conducted in accordance with the Declaration of the World Medical Association, and was reviewed and approved by the Seoul National University Hospital Institutional Review Board (IRB no. 1605–028-760). Because of the study’s retrospective design, the need for consent to participate was waived.

### Measures

Demographic and medical data, including age, Gower sign results, and dystrophin gene study results, were collected. Clinical variables, such as lower extremity joint passive ROM and sagittal plane contracture angles (flexion/extension movements), were recorded. The contracture angles were measured bilaterally in the hip, knee, and ankle joints, using a goniometer, during clinical examinations performed by a physical therapist. Goniometry is the most commonly used technique for measuring joint motion limitations due to muscle contracture. With the patients in a supine position, the same physiotherapist measured and recorded all lower extremity joint passive ROMs, according to the method of Norkin and White [11]. Because the same physiotherapist performed all of the joint contracture measurements, the intra-tester measurement reliability is likely high, according to Pandya et al. [12]. For each measurement, the protocol guided the reference points for the fulcrum and the proximal and distal arms of the goniometer. At each time point, duplicate passive ROM measurements were obtained and averaged.

A physician recorded the Vignos Scale score to grade lower limb function of the children with DMD [13]. The Vignos Scale classifies patients with DMD into 10 categories, based on their ability to walk. Patients with Vignos Scale scores of 1–7 were considered ambulatory, whereas patients with scores of 8–10 were considered non-ambulatory.

### Procedure

The severities of hip flexion (HF), knee flexion (KF), and ankle plantarflexion (APF) contractures were classified, based on a previous study [9], as mild (1°–19°), moderate (20°–40°), and severe (> 40°) for HF contractures; mild (1°–14°), moderate (15°–40°), and severe (> 40°) for KF contractures; and mild (1°–14°), moderate (15°–30°), and severe (> 30°) for APF contractures.

We investigated whether significant differences in passive stretching exercise participation existed between ambulatory and non-ambulatory patients. All patients with DMD who participated in the stretching exercises received help from their physical therapist at hospital. Children with DMD who performed stretching exercises for > 5 min/session, for > 3 sessions/week, were categorized into the stretching group; the others were classified into the non-stretching group.

### Data analysis

Means and standard deviations were used to describe the basic patient characteristics. The mean values for right and left HF, KF, and APF angles were obtained for each patient, and the HF, KF, and APF contracture angles for both legs were averaged; *t*-tests confirmed the absence of significant differences between the left and right legs for all measurements. Data were also stratified according to patients categorized as ambulatory or non-ambulatory.

A generalized estimating equation was used to assess the extent of the differences in the degree of lower extremity joint contractures, depending on ambulatory status, and the degree of lower extremity joint contracture, between joint sites, depending on the patients’ ambulatory status. The relationship among the HF, KF and APF contracture angles were assumed to be constant. The mean contracture angle of each lower extremity joint was compared to that of the hip joint. The *p*-values and 99.3% confidence intervals, including the Bonferroni correction, are presented for all seven tests.

Within each group, the mean HF, KF, and APF joint contracture angles were analyzed, using an independent samples *t*-test, to test for differences between the stretching and non-stretching groups.

Statistical analyses were performed using SPSS version 21.0 (IBM, Armonk, NY, USA). The level of statistical significance was set at 5% for 2-tailed tests.

## Results

### Demographic and clinical information

**Fig. 1:**
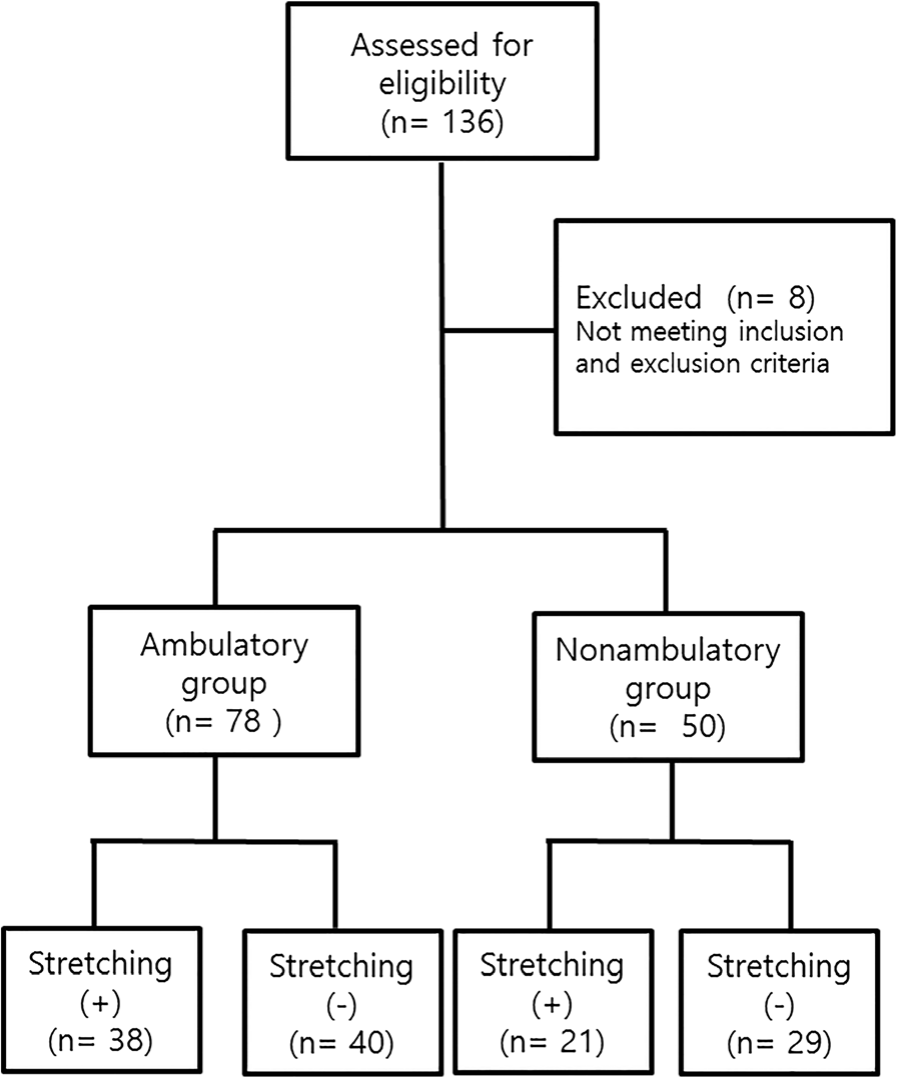
Study flow diagram

A total of 136 boys with DMD were evaluated for inclusion in the study; eight were excluded due to not meeting the study criteria (Fig. 1). Of the 128 eligible patients, 78 (61%) were included in the ambulatory group and 50 (39%) were in the non-ambulatory group. The mean age in the ambulatory group was 9.1 ± 2.2 years (range, 4–15 years) and that in the non-ambulatory group was 13.3 ± 3.0 years (range, 8–23 years); none of the patients withdrew from corticosteroid therapy. The number of patients based on ambulatory ability, is shown in Table 1.

**Table 1:** Patient Vignos Scale score

Vignos score	Number (%)
1	19 (14.8)
2	12 (9.4)
3	22 (17.2)
4	11 (8.6)
5	4 (3.1)
6	5 (3.9)
7	5 (3.9)
≥8	50 (39.1)
Total	128 (100)

### Lower extremity joint contracture comparisons

Table 2 shows the number of patients exhibiting each lower extremity joint contracture angle severity. In the ambulatory group, HF and KF contractures were observed infrequently, whereas APF contractures were observed more frequently in ambulatory; 41 patients (52.6%) in the ambulatory group showed APF contractures.

**Table 2:** Severity of lower limb joint contractures in patients with DMD, based on ambulatory status

	No contracture	Mild	Moderate	Severe	Total
Hip flexion					
Ambulatory	70 (89.7)	7 (9)	1 (1.3)	0 (0)	78 (100)
Non-ambulatory	7 (14.0)	17 (34.0)	24 (48.0)	2 (4.0)	50 (100)
Knee flexion					
Ambulatory	61 (78.2)	13 (16.7)	3 (3.8)	1 (1.3)	78 (100)
Non-ambulatory	2 (4.0)	3 (6.0)	19 (38.0)	26 (52.0)	50 (100)
Ankle plantarflexion					
Ambulatory	37 (47.4)	30 (38.5)	10 (12.8)	1 (1.3)	78 (100)
Non-ambulatory	1 (2.0)	8 (16.0)	14 (28.0)	27 (54.0)	50 (100)

*DMD* Duchenne muscular dystrophy; values are presented as n (%)

The interactions were significant, based on the use of the generalized estimating equation analysis. Consequently, the contracture angles of each lower extremity joint were compared based on ambulatory status. The mean KF and APF contracture angles were compared with the mean HF contracture angle in both the ambulatory and non-ambulatory groups. The mean APF contracture angle was 4.9^o^ greater (99.7% confidence interval, 2.6–7.3) than the mean HF contracture angle (adjusted *p* < .0001), in the ambulatory group. Comparing the ambulatory and non-ambulatory groups, the mean HF angle was 22.0^o^ (99.7% confidence interval, 17.5–26.5) greater, the mean KF contracture angle was 40.4^o^ (99.7% confidence interval, 30.6–50.2) greater, and the mean APF contracture angle was 28.1^o^ greater (99.7% confidence interval, 19.7–36.5) in the non-ambulatory group than in the ambulatory group (Table 3).

**Table 3:** Average lower extremity joint contracture angles, estimated using a generalized estimating equation

Ambulatory status	Joint	Estimated average values (confidence interval)	*p*-value^*^	*p*-value^**^
Ambulatory	Hip	1.5 (0.5–2.5)		<.0001
	Knee	3.1 (1.6–4.7)	.092	<.0001
	Ankle	6.4 (4.6–8.2)	<.0001	<.0001
Non-ambulatory	Hip	23.5 (20.4–26.6)		
	Knee	43.5 (36.5–50.4)	<.0001	
	Ankle	34.5 (28.6–40.3)	.001	

^*^Corrected *p*-values for comparisons of other ankle and knee joint contracture angles with that of the hip joint

^**^Corrected *p*-value for comparisons of the joint contracture angles, depending on ambulatory status

### Differences in joint contracture angles, based on stretching participation

The mean age of the ambulatory boys with DMD in the stretching group was 8.8 ± 2.2 years and 9.4 ± 2.2 years in the non-stretching group. Among the non-ambulatory boys with DMD, the mean age was 13.6 ± 3.8 years in the stretching group and 13.1 ± 2.4 years in the non-stretching group. There were no significant differences in the mean ages of the boys, based on ambulatory ability, in the stretching and non-stretching groups. The mean duration of each stretching session was 16.2 ± 8.8 min (range, 5–30 min) for the ambulatory group and 16.8 ± 13.4 min (range, 5–60 min) in the non-ambulatory group; 31 (81.6%) patients in the ambulatory group and 17 (81.0%) in the non-ambulatory group had performed stretching exercises for > 1 year. No differences were seen in the severities of the HF, KF, and APF joint contractures between the stretching and non-stretching groups, regardless of ambulatory status (Table 4).

**Table 4:** Independent-sample *t*-test results comparing the stretching and non-stretching groups

	Ambulatory group				Non-ambulatory group		
	Joint contracture				Joint contracture		
	Hip flexion	Knee flexion	Ankle plantarflexion		Hip flexion	Knee flexion	Ankle plantarflexion
Stretching (*n* = 38)	1.4 ± 4.3	2.4 ± 5.0	5.9 ± 6.4	Stretching (*n* = 21)	20.0 ± 11.5	37.4 ± 25.1	31.5 ± 22.1
No stretching (*n* = 40)	1.5 ± 4.9	3.8 ± 8.6	6.9 ± 9.6	No stretching (*n* = 29)	26.0 ± 10.8	47.9 ± 25.0	36.6 ± 20.9
*p*-value	.92	.39	.60	*p*-value	.06	.15	.41

Values are presented as means ± standard deviations

## Discussion

This study examined HF, KF, and APF contractures among male patients with DMD, based on the patients’ ambulatory status, and investigated the differences in major joint contractures, based on passive stretching exercise participation. Our findings indicated that HF, KF, and APF contractures are more common and severe when there is deterioration of ambulatory function. Moreover, stretching exercises alone are unlikely to prevent lower extremity joint contractures.

The frequency and severity of lower extremity joint contractures rapidly increases after the loss of ambulatory function in children with DMD. This finding is consistent with the results of a previous study by McDonald et al. [9]. However, unlike their results, lower extremity contractures were common in the ambulatory patients in our study, especially those involving the ankle joint. In ambulatory patients, APF contractures may be associated with a compensatory mechanism. Gaudreault et al. reported that, in children with DMD, the APF moments caused by early APF contractures contribute to the production of the net APF moment during the stance phase of gait [14]. An alternative hypothesis, suggested by Sutherland et al., is that as weakness progresses, ambulatory DMD patients exert APF moments during the stance phase of gait to oppose KF moments, allowing movement of the force line in front of the knee joint’s center. They suggested that this compensatory gastrocnemius-soleus muscle group activity assists with knee stability as well as the muscle imbalance contributed by the APF contracture [15]. However, when APF contracture proceeds further, knee joint compensation and increased HF angle during the stance phase may decrease gait stability [16, 17]. Additionally, excessive APF contractures often make the wearing of regular shoes and passive standing difficult for patients with DMD, after the loss of ambulatory function. Because this study was a cross-sectional study, a demonstration of the interplay between APF contracture and muscle weakness that occurs during disease progression was not possible.

Most previous studies on joint contractures in patients with DMD were reported before universal steroid use. In some studies, those who received steroid medications were excluded from the analysis. Unlike previous studies, all of the patients in the present study were prescribed deflazacort. However, our results are similar to those obtained when steroids were not widely used [9]. Mendell et al. [18] also suggested that although prednisone treatment improves the strength and functioning of patients with DMD, it has no significant effect on major joint ROM.

No differences were observed in the contracture angles between the stretching and non-stretching groups, regardless of ambulatory status. These results are comparable with the results of Brooke et al. [5], who reported no correlation between patients performing passive joint stretching exercises and joint contractures. A few authors have suggested that joint angles should be managed in parallel with orthotic management, rather than solely through stretching. Hyde et al. [19] demonstrated that patients undergoing passive stretching combined with the use of nighttime splints demonstrated 23% less annual change in APF contracture angles than did the patients managed with stretching only. Others [20] performed a longitudinal analysis that suggested early and continuous use of daytime articulated AFOs promoted positive changes in the kinematic parameters of gait when used before the functional deficit became too advanced, alleviating the increase in pelvic tilt and decrease in hip extension angles during the stance phase of gait. Standing devices might also be considered for use, with or without AFO application, because they allow maintenance of an upright position and provide leg joint stretching [21].

Because of the limited evidence available regarding the optimal frequency of stretching exercises in children with DMD, we arbitrarily set the criteria for the stretching exercise group as > 3 sessions/week; in clinical situations, daily stretching exercises may not be realistic. Further, the current stretching recommendations from the American College of Sports Medicine apply only to healthy adults. To maintain long-term flexibility and ROM, stretching exercises are recommended at least 2–3 days per week, and the same recommendations for stretching exercises are usually applied to pediatric populations, based on these recommendations [22]. Based on a systematic review on ankle dorsiflexion stretching [23], calf muscle stretching provides increased ankle dorsiflexion, particularly after 5–30 min of stretching in healthy individuals. Therefore, children who stretched > 5 min/session were included in the stretching group, in this study.

We acknowledge that this study has several limitations. First, the cross-sectional design may effect selection and institutional biases. Second, the duration and technical appropriateness of the stretching exercises were not controlled. Although the patients and their parents received stretching technique education from the institution where the study was conducted, the stretching techniques were not monitored or evaluated. Future prospective studies that control the quality and quantity of this type of intervention could help better understand its benefits for delaying the progression of joint contractures.

## Conclusion

HF, KF, and APF contractures were more common and more severe in non-ambulatory patients. APF contractures were observed more frequently, even early in the ambulatory period. Prevention of lower extremity joint contractures solely through stretching exercises is unlikely. Knowledge of lower extremity joint contracture profiles, based on ambulatory status, may be useful for developing appropriate strategies for joint management in this patient group.

## Abbreviations

APF: Ankle plantarflexion
DMD: Duchenne muscular dystrophy
HF: Hip flexion
KF: Knee flexion
ROM: Range-of-motion
